# Macronutrients and Micronutrients in Parenteral Nutrition

**DOI:** 10.3390/nu17010046

**Published:** 2024-12-27

**Authors:** Giovanna Verlato, Marta Meneghelli, Maria Elena Cavicchiolo

**Affiliations:** 1 Neonatal Intensive Care Unit (NICU), Department of Women’s and Children’s Health, University Hospital of Padova, 35128 Padova, Italy; marta.meneghelli@aopd.veneto.it (M.M.); mariaelena.cavicchiolo@aopd.veneto.it (M.E.C.); 2 Paediatric Nutrition Service, NICU—Department of Women’s and Children’s Health, University Hospital of Padova, 35128 Padova, Italy

Appropriate nutrition is of paramount importance during infancy and childhood, and Parenteral Nutrition (PN), which is the intravenous infusion of nutrients in the elementary form, may be necessary as a supplement or a full replacement for enteral nutrition. PN was developed in the 1960s, and since then, it has become a life-saving procedure for individuals who are unable to gain all the macro- and micronutrients they need through the digestive system; for this reason, it is essential to continuously improve the quality of PN components and to study the optimal intake of PN macro- and micronutrients, particularly for newborn’s and children’s health, along with the prevention of the possible side effects of long-term PN.

PN is used when impaired gastrointestinal function (intestinal insult, inflammation, obstruction, malabsorption, dysmotility) occurs, in extraintestinal illnesses leading to malnutrition that cannot be entirely prevented by the enteral route and in intestinal immaturity, as occurs in preterm newborns. Therefore, in this Special Issue, we first focus on the use of PN in preterm newborns.

Preterm birth, which is defined as a birth that occurs before 37 weeks of gestation, is known to be on the rise globally (around 10–12%) because of improvements in pregnancy and neonatal care and in nutritional interventions shortly after birth. Adequate nutrition is necessary to meet the increased metabolic demands of the preterm newborn since cumulative nutritional deficiencies occurring early in life may have both short-term and long-term effects and determine not only growth failure but also increased susceptibility to infections, increased severity of postnatal diseases, and adverse neurodevelopment outcomes.

Nonetheless, complete enteral feeding is frequently delayed in premature infants for gut immaturity and unstable clinical conditions, so PN represents an essential therapeutic option for these newborns early in life.

As reported in the work by Rizzo et al. [1], available recommendations suggest starting PN as soon as possible after birth and rapidly ensuring adequate intake using a well-balanced solution; a minimum intake of 45–55 Kcal/kg/day on the first day of life, with at least 1.5 g/kg/day of amino acids, 1 g/kg/day of lipids (or providing at least 0.25 g/kg/day of linoleic acid depending on the lipid emulsions used) and sufficient minerals, vitamins and trace elements, is now recommended in all preterm newborns [2,3,4,5]. Intake should be increased during the first week of life, according to the clinical condition, up to 90–120 Kcal/kg/day, with 2.5–3.5 g/kg/day of amino acids and 3–4 g/kg/day of lipid aiming, after the initial postnatal nadir of weight loss, ensuring weight gain of 17–20 g/kg/day. Nonetheless, we must also keep in mind the fact that extremely preterm newborns and small-for-gestational-age newborns are at risk of refeeding syndrome, and we need to monitor and replace phosphate and potassium if values are low.

In addition, preterm newborns, particularly those in critical conditions, may exhibit reduced tolerance to parenteral macronutrient intake, with hyperglycemia being linked to negative outcomes [6].

For this reason, Boscarino et al. analyzed the effects of hypertriglyceridemia and hyperglycemia related to PN on respiratory outcomes and neurodevelopment (Bayley Scale III at 24 months) in surviving preterm infants with a gestational age at birth < 32 weeks or birth weight < 1500 g [7,8]. The studies suggest that hypertriglyceridemia and hyperglycemia may have mid-term (duration of invasive mechanical ventilation) and long-term (motor delay at 24 months) consequences in preterm newborns. Nonetheless, the author’s conclusion is that further randomized controlled trials are urgently needed to confirm these results.

Interestingly, Fivez et al. demonstrated, in 1440 critically ill children (term newborns to 17 years old children) during the first week of admission in the Intensive Care Unit, that permissive underfeeding with PN is safe, although the study did not include preterm newborns [9]. Of note, the European guidelines did not support a change in the current practice regarding preterm infants and suggested starting PN immediately to cover basic macronutrient needs and, at the same time, avoid either hypo- or hypernutrition.

In this Special Issue, we focus then on the importance of PN in specific diseases such Necrotizing Enterocolitis (NEC). 

Guiducci et al. [10], in their narrative review, outlined that PN should start as soon as possible during the acute phase, with careful fluid management to prevent electrolyte abnormalities and fluid overload. The consumption of macronutrients (protein, glucose, and lipids) should be tailored to each stage of the illness using a composite lipid emulsion. Careful vitamin and trace element monitoring, beginning during the recovery phase, should be performed in particular for patients who need long-term PN to sustain growth and prevent long-term deficits or overload with consequent negative effects.

Furthermore, it is important to thoroughly evaluate the biochemical and clinical indicators of PN tolerance, as well as, in case of long-term PN, the associated morbidities (i.e., growth failure, metabolic bone disease and intestinal failure-associated liver disease). Although NEC is becoming the main cause of short bowel syndrome (SBS) in patients of pediatric age, this unique population has not received much attention in research; therefore, more research is necessary to enhance nutritional strategies and manage long-term effects in these infants.

The long-term consequences of prolonged PN is the topic of the study by Gatti et al. [11], who performed a systematic review to determine risk factors for the development of metabolic bone disease (MBD) and the prevalence of the condition in children with intestinal failure (IF). Up to 45% of children with IF that needed long-term PN had reduced bone mineral density, which was associated with age, growth failure, and particular IF etiologies, despite the fact that the frequency and criteria of MBD widely varied.

Research shows that even when PN dependency is treated, children with IF should receive ongoing follow-up treatment with frequent analysis of bone status. Prognostic and therapeutic views will be the main foci of longitudinal prospective research to allow us to better identify the etiology and effects of MBD.

In conclusion, adequate PN can support the primary goal of feeding, which is maximizing children’s lifelong health, well-being, and quality of life by allowing them to reach their full genetic growth potential when enteral nutrition alone is insufficient.

Established guidelines are available; nonetheless, tailored macro- and micronutrient intakes must be provided for avoiding either hypo- or hypernutrition. We need to be aware of the long-term deficits and morbidities associated with long-term PN and take all the necessary steps to prevent and treat them as soon as possible. This argument requires prospective population multicenter research; in particular, longitudinal, prospective pediatric studies should be conducted to evaluate the impacts of treatment regimens and the influence of individual PN components on child’s health.
